# Identification and manipulation of *Neurospora crassa* genes involved in sensitivity to furfural

**DOI:** 10.1186/s13068-019-1550-4

**Published:** 2019-09-04

**Authors:** Daria Feldman, David J. Kowbel, Adi Cohen, N. Louise Glass, Yitzhak Hadar, Oded Yarden

**Affiliations:** 1 Department of Plant Pathology and Microbiology, The R.H. Smith Faculty Agriculture, Food and Environment, The Hebrew University of Jerusalem, 7600001 Rehovot, Israel; 2 Department of Plant and Microbial Biology, University of California, Berkeley, Berkeley, CA 94720 USA; 3 Environmental Genomics and Systems Biology Division, The Lawrence Berkeley National Laboratory, 1 Cyclotron Road, Berkeley, CA 94720 USA

**Keywords:** Furfural, *Neurospora crassa*, Furan, Pretreatment, CRE1, Aldehyde dehydrogenases

## Abstract

**Background:**

Biofuels derived from lignocellulosic biomass are a viable alternative to fossil fuels required for transportation. Following plant biomass pretreatment, the furan derivative furfural is present at concentrations which are inhibitory to yeasts. Detoxification of furfural is thus important for efficient fermentation. Here, we searched for new genetic attributes in the fungus *Neurospora crassa* that may be linked to furfural tolerance. The fact that furfural is involved in the natural process of sexual spore germination of *N. crassa* and that this fungus is highly amenable to genetic manipulations makes it a rational candidate for this study.

**Results:**

Both hypothesis-based and unbiased (random promotor mutagenesis) approaches were performed to identify *N. crassa* genes associated with the response to furfural. Changes in the transcriptional profile following exposure to furfural revealed that the affected processes were, overall, similar to those observed in *Saccharomyces cerevisiae*. *N. crassa* was more tolerant (by ~ 30%) to furfural when carboxymethyl cellulose was the main carbon source as opposed to sucrose, indicative of a link between carbohydrate metabolism and furfural tolerance. We also observed increased tolerance in a Δ*cre*-*1* mutant (CRE-1 is a key transcription factor that regulates the ability of fungi to utilize non-preferred carbon sources). In addition, analysis of aldehyde dehydrogenase mutants showed that *ahd*-*2* (NCU00378) was involved in tolerance to furfural as well as the predicted membrane transporter NCU05580 (*flr*-*1*), a homolog of *FLR1* in *S. cerevisiae*. Further to the rational screening, an unbiased approach revealed additional genes whose inactivation conferred increased tolerance to furfural: (i) NCU02488, which affected the abundance of the non-anchored cell wall protein NCW-1 (NCU05137), and (ii) the zinc finger protein NCU01407.

**Conclusions:**

We identified attributes in *N. crassa* associated with tolerance or degradation of furfural, using complementary research approaches. The manipulation of the genes involved in furan sensitivity can provide a means for improving the production of biofuel producing strains. Similar research approaches can be utilized in *N. crassa* and other filamentous fungi to identify additional attributes relevant to other furans or toxic chemicals.

## Introduction

Biofuels derived from lignocellulosic biomass are a viable alternative to fossil fuels required for transportation [1]. In second-generation biofuels, pretreatment of plant biomass using a thermo and/or chemical process is necessary to make carbohydrates available for enzymatic hydrolysis and fermentation by disrupting their linkage to lignin in the plant cell wall [1, 2]. The beneficial effects of pretreatment of lignocellulosic materials have been recognized for a long time, and the goal of the process is to remove lignin, reduce the crystallinity of cellulose, and increase the porosity of the lignocellulosic materials [1, 3, 4].

The pretreatment processes, using dilute acid and wet oxidation, are accompanied by the production of inhibitory compounds, particularly furans, that can inhibit efficient sugar utilization by fermentation organisms, mainly *Saccharomyces cerevisiae* [3, 5–7], thus imposing a rate-limiting step in production. Two of the furan derivatives generated during these pretreatment are 5-hydroxymethyl-2-furaldehyde (HMF) and furfural, which are the most potent microbial inhibitors that are formed by dehydration of hexoses and pentoses, respectively [8–11]. The quantities of furans produced vary based on the type of raw material used and the pretreatment procedure employed. Furfural is usually found at lower levels than HMF. However, it is often present at a sufficient concentration (~ 1 g/l) to be inhibitory [3, 12, 13].

Furfural has been studied intensively and its conversion to furfuryl alcohol (furan methanol) and further reduction to 2-furoic acid by *S. cerevisiae* has been demonstrated [14, 15]. After exposure to furfural, *S. cerevisiae* proteins involved in glucose fermentation and the tricarboxylic acid cycle were found to be more abundant in the exposed cells than in the untreated controls, while the abundance of proteins involved in glycerol biosynthesis were reduced. The aldehyde dehydrogenases Adh5p and Adh1p were suggested to be the catalytic agents for furfural reduction [16]. ^13^C-labeled metabolic flux and transcriptional analyses showed that ADH7 and YKL071W (NADH-dependent aldehyde reductase) are associated with yeast resistance to furfural [17]. Another important element in mediating yeast tolerance is the pleiotropic drug resistance (PDR) family, which function as ATP binding and chemical resistance agents. Genes encoding PDRs display a consistent increase in expression when *S. cerevisiae* is exposed by furfural and HMF, promoting cellular survival and adaptation to the inhibitor-induced stress [18–20].

Research on the filamentous fungus *Neurospora crassa* benefits from a large toolbox of molecular, genomics, and cytological techniques that are unavailable for most other filamentous fungi [21–23]. These assets include a near-full genome deletion strain set of ~ 12,500 mutants [24]. In nature, *N. crassa* grows on the stems of grasses, e.g., sugarcane that has been recently killed by burning [25–28]. In recent years, the ability of *N. crassa* to grow on plant material became the focus of studies that revealed that under such conditions the expression and secretion of a large number of cellulases, hemicellulases and proteins of unknown function are induced [29]. Thus, *N. crassa* has also become an important organism relevant to understanding plant cell wall degradation by fungi and, subsequently, elucidating some of the potential mechanisms that can be exploited for increasing biofuel yield [30–34]. It was also shown that furfural, produced during burning of plant substrates, can function as an inducer of sexual spore germination in *N. crassa* [25, 35, 36]. In addition, a decrease in concentrations of furfural occurred during fermentation of plant biomass by *N. crassa* [37]. These studies suggest that *N. crassa* can cope with furfural by the expression of unique genes or pathways that, once identified, can be potentially exploited by expression in a heterologous manner in fermentation organisms.

The aim of this study was to uncover new genetic attributes in *N. crassa* involved in conferring tolerance to furfural, using complementing experimental approaches. Based on RNA-seq data analyses, an aldehyde dehydrogenase *ahd*-*2* (NCU00378) and the transcription factor responsible for carbon catabolite repression, *cre*-*1*, were identified to be involved in tolerance to furfural. Using a hypothesis-driven approach and based on screening relevant known homologs from other organisms, a predicted membrane transporter (NCU05580) was identified as relevant to furfural sensitivity. Lastly, using an unbiased approach, in which a tagged promotor cassette was randomly integrated into the fungal genome, we determined that inactivation of two hypothetical proteins, NCU02488 or NCU01407, resulted in increased tolerance of *N. crassa* to furfural.

## Results

### Developmental and physiological responses of *N. crassa* to furfural

#### The effect of furfural on growth rate

The toxic effect of different furfural concentrations on the relative growth rate of *N. crassa* was measured. Furfural affected conidial germination and subsequent colony development in a dose-dependent manner with an IC_50_ ~ 12.5 mM, as measured 20 h post inoculation (Additional file 1: Figure S1A). The inhibitory value we measured was similar to the IC_50_ of 16 mM measured for *S. cerevisiae* [38].

**Fig. 1:**
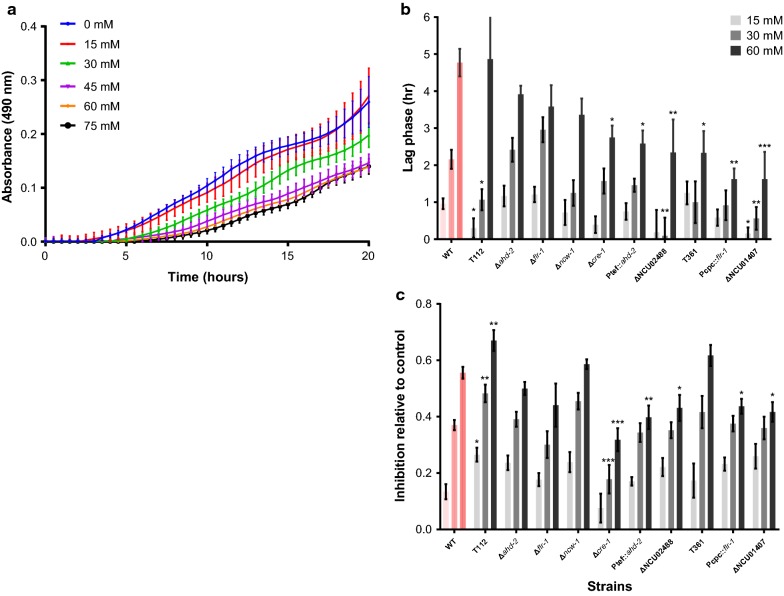
Effect of furfural on growth of *N. crassa* wild-type and mutant strains in a 96-well plate assay, as measured at 490 nm. **a** Wells of a 96-well plate were inoculated with 4 × 10^5^ conidia of the wild-type strain in VgS media supplemented with different concentrations (15–75 mM) of furfural. **b** Delay in lag phase as expressed in the time required for detectable initiation of culture growth, compared to the control lacking furfural (15, 30 and 60 mM). **c** Inhibition in relative growth, as determined by O.D. of cultures exposed to furfural (15, 30 and 60 mM) relative to that of the control, 20 h after inoculation. Asterisks in **b**, **c** indicate a significant difference between the mutant strains and wild-type subjected to the same treatment (*P < 0.05, **P < 0.01, and ***P < 0.001 by Student’s *t* test). The wild-type control is shown in pink. The values represent the average of at least six biological replicates

To further monitor the effect of furfural on colony growth, we used a high throughput 96-well plate system. Liquid medium was inoculated with conidia of *N. crassa* and supplemented with different concentrations of furfural (15–75 mM) (Fig. 1a). Two main parameters were measured to assess the inhibitory effects: (i) the lag phase, referring to the time required for initiation of culture biomass accumulation as compared to the control lacking furfural, and (ii) the inhibition in relative growth rate, as determined by measuring the O.D. of cultures exposed to furfural compared to that of the control 20 h after inoculation. We focused on the 15, 30 and 60 mM furfural treatments, which imposed a lag period in colony development of 0.96, 2.16 and 4.77 h, as compared to untreated controls (Fig. 1b). Growth inhibition (as determined at the 20 h time point) also correlated to the furfural concentration, resulting in 12, 25 and 57%, at the respective furfural concentrations (Fig. 1c).

#### Furfural inhibits conidial germination

**Fig. 2:**
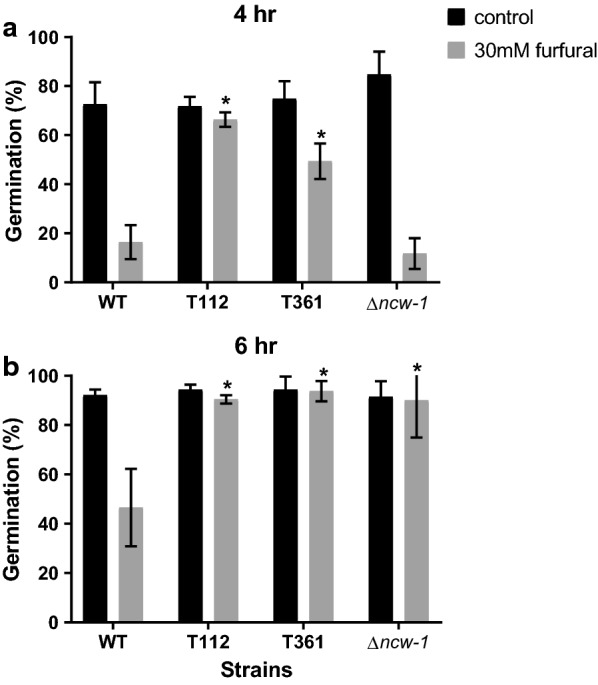
Conidial germination of wild-type and mutant strains as affected by exposure to furfural. Conidial germination of wild type, T112, T361 and ∆*ncw*-*1*, 4 (**a**) and 6 (**b**) h after inoculation. Asterisks in the figure indicate a significant difference between the mutant strains and wild type subjected to the same treatment (*P < 0.05, by Student’s *t* test). The values represent the average of three biological replicates

We hypothesized that the extended lag phase in the 96-well screen was due to a delay in conidial germination. To address this possibility, *N. crassa* conidia were inoculated into medium amended with a high dose of furfural (60 mM). The growth of the fungus was completely inhibited during the first 40 h, after which it recovered and the growth was inhibited by only ~ 36% (natural degradation of furfural was negligible) (Additional file 1: Figure S1B), supporting the occurrence of increased toxicity during germination. This result led us to further explore the effect of the compound on conidial germination. We monitored the germination rate of conidia 4 and 6 h after inoculation, after which ~ 75% and ~ 90% of the control conidia germinated at these two time points, while only 12% and 47% of the conidia exposed to 30 mM furfural did so (Fig. 2). These data suggest that the major effect of furfural was in delaying asexual spore germination.

#### Furfural is similarly reduced by either NADH or NADPH

**Fig. 3:**
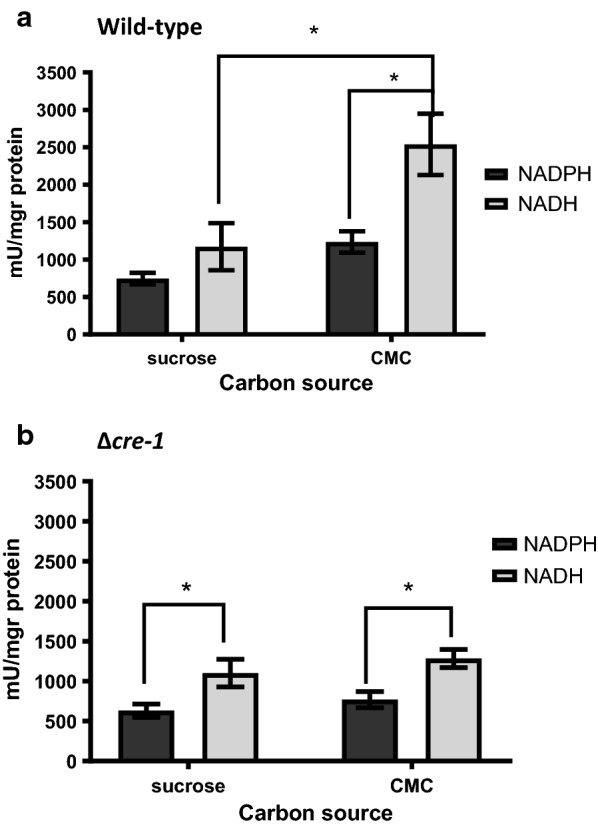
In vitro depletion of furfural is dependent on carbon catabolic repression. Depletion of NADPH or NADH was monitored *in vitro* with free-cell extracts of *N. crassa* wild-type (**a**) or Δ*cre*-*1* (**b**) strains with 10 mM furfural as a substrate. The cultures were grown on Vogels media with 1.5% sucrose or CMC as a carbon source for 16 h, before proteins were extracted. Asterisks in the figure indicate a significant difference (*P < 0.05, by Student’s *t* test). The values represent the average of three biological replicates

In *S. cerevisiae*, intracellular furfural is reduced within the intracellular fraction by either NADPH or NADH as co-factors [14, 20, 39]. When we monitored the ability of *N. crassa* cell-free extracts to reduce furfural in vitro, and specific activity as measured by NAD(P)H depletion, we found a similar effect with both co-factors (Fig. 3a). These results suggest that the intracellular enzymes involved in the reduction were not specific and included those that can utilize either NADH or NADPH. Thus, it is possible that several different cellular pathways may be involved in modification of furfural.

### Changes in gene expression following exposure to furfural

**Fig. 4:**
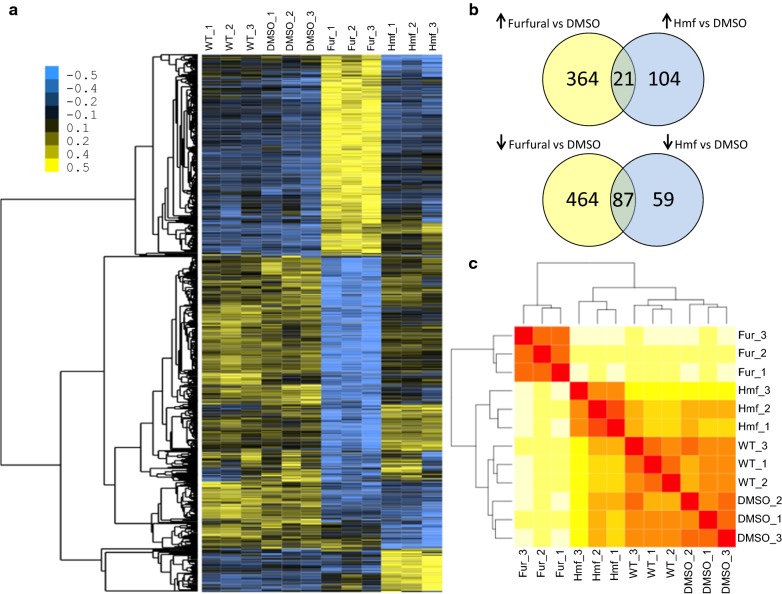
Summary of gene expression in *N. crassa* following exposure to furfural. Each sequence read was mapped to the *Neurospora* genome v12 using tophat v2.04 [67] and the expression of each gene was normalized as FPKM using cufflinks v2.02 [68] with a cutoff value of log2 ≥ 1.0 and an FDR-adjusted *P* value of ≤ 0.01. **a** Hierarchical clustering was performed on expression data from samples with cluster3.0 with blue representing low expression and yellow representing high expression. **b** Venn diagram of significantly differentially expressed genes in *N. crassa* following exposure to furfural and HMF. **c** All RNA-seq data sets were clustered by Euclidean distance using a variance-stabilizing transformation module in DEseq v1.14 [82]. Darker colors represent higher similarity between replicates

RNA-seq was performed to assess the genetic response of *N. crassa* to furfural and to identify potential targets for subsequent gene manipulation experiments. *N. crassa* was exposed to 10 mM furfural for 4 h, to achieve 35% inhibition in biomass accumulation as compared to the control. Total RNA isolated and used to construct RNA-seq libraries was analyzed using hierarchical clustering (Fig. 4a) and clustered by Euclidean distance (Fig. 4c). The overall summary of the RNA-seq analysis is shown in Additional file 2: Table S1. Following exposure to furfural, an increase in the expression levels of 385 genes was observed, while that of 551 genes was reduced (Fig. 4b). Parallel to the analysis of the transcriptional response to the presence of furfural, we also performed RNA-seq analyses of cultures exposed to HMF, another inhibitory furan produced during pre-treatment with dilute acid or wet oxidation. Exposure to 10 mM of HMF was accompanied by increased differential expression of 125 genes, and a reduction in that of 146 genes (Fig. 4b).

Biological processes affected by exposure to furfural (*P* value < 0.01) included: translation, transcription, transmembrane transport, alpha-amino acid biosynthesis, oxidation–reduction processes, mitochondrial organization, carbohydrate metabolic processes, transport, and cellular amino acid metabolic processes. Notable genes whose expression levels were increased following exposure to furfural included alternative oxidase 1 (NCU07953) and genes encoding enzymes in branched-chain amino acid synthesis (NCU09864, NCU03913 and NCU02704 (Additional file 1: Table S2).

The largest child GO term associated with genes that showed increased expression levels upon exposure to furfural was “oxidation–reduction process” (GO:0055114) with 53 genes. The genes with at least a fourfold increase in FPKM (fragments per kilobase of unique exons per million mapped reads in library) encode a predicted trichothecene C-15 hydroxylase (NCU00732), an alternative oxidase-1 (NCU07953), a pentachlorophenol monooxygenase (NCU04591), a phosphoenolpyruvate carboxykinase (NCU09873), a homogentisate 1,2-dioxygenase (NCU05499), a monooxygenase (NCU08747), a 4-hydroxyphenylpyruvate dioxygenase (NCU01830), hypothetical proteins (NCU02031 and NCU09165), an NADPH-adrenodoxin reductase (NCU08005), a malate synthase (NCU10007), a cytochrome B5 (NCU08060), and a isovaleryl-CoA dehydrogenase (NCU02126) (Additional file 3: Table S9).

**Fig. 5:**
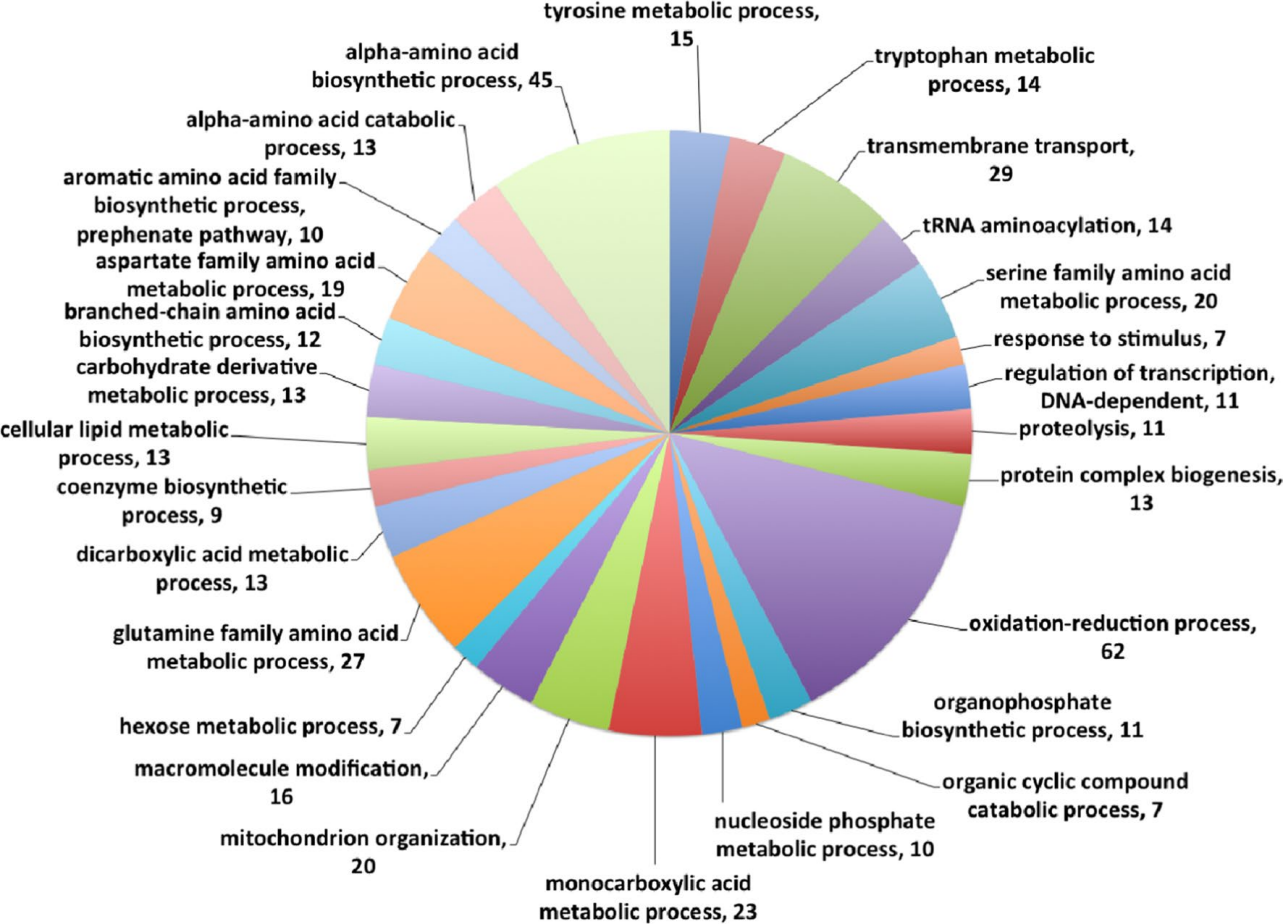
GO terms associated with biological processes affected upon exposure to furfural in *N. crassa.* GO terms were associated with all protein coding genes using the program Blast2Go v 2.8 [70] and a multilevel pie chart generated from genes that are up regulated with a log2 value ≥ 1.0 and *P*-adjusted value ≤ 0.01 and a node score > 6.0. Only terminal nodes are presented in the pie chart with the node score for each biological process

A one-tailed Fisher’s exact test was used to assess which GO terms were significantly enriched by exposure to furfural (Fig. 5 and Additional file 3: Table S9). A number of processes associated with amino acid metabolism showed significant enrichment, including “alpha-amino acid metabolism” (adjusted *P* value 5.9e−13), “aspartate family amino acid metabolism” (adjusted *P* value 4.1e−4) and “glutamine family amino acid metabolism” (adjusted *P* value 3.3e−4). In addition, “tRNA aminoacylation”, which is necessary for t-RNA ligation to its specific amino acid during translation, was enriched (adjusted *P* value 7.1e−7). Exposure to furfural significantly affected the expression of genes in the aspartate family, including isoleucine, threonine, lysine, methionine and asparagine and the glutamine family, and proline and glutamate amino acid biosynthesis pathways. The GO term for “oxidation–reduction processes” was also significantly enriched (adjusted *P* value 4.6e−4).

Genes that showed a significant decrease in expression upon exposure to furfural included structural ribosomal constituents (adjusted *P* value 7.2e5) and all encoded ribosomal proteins. Forty-three genes associated with “carbohydrate metabolic processes” (adjusted *P* value 7.2e-5) also showed significant downregulation by exposure to furfural. Three genes that showed significant downregulation (at least fourfold) included *ncw*-*1* (NCU05137), encoding a non-anchored cell wall protein [40] and 1,3-beta-glucanosyltransferase genes (NCU07253 and NCU08909), which encode predicted cell wall proteins in *N. crassa* [41].

We screened 13 deletion mutants of genes that had the highest up- or downregulation in response to furfural from the *Neurospora* deletion collection, which were obtained from the FGSC (Fungal Genetics Stock Center) [42]. Seven mutants contained a deletion of genes that were upregulated in response to furfural (NCU04697, NCU00732, NCU07953, NCU04591, NCU05045, NCU08561 and NCU08096), and five mutants containing a deletion of genes that were downregulated in response to furfural (NCU08907, NCU08720, NCU08457, NCU06912 and NCU00399). Six additional mutants, each containing a deletion of one of six genes that were significantly affected by the presence of HMF, were also screened for sensitivity to HMF. All the deletion strains exhibited a similar tolerance to either furfural or HMF as the wild-type parental strain (Additional file 1: Table S3). Overall, while RNA-seq has provided a metabolic overview of the fungal transcriptome in response to the presence of furfural, the functional contribution of genes was not apparent by a standard single-gene reverse genetics approach.

### Attributes of furfural tolerance identified by RNA-seq analyses

#### Carbohydrate metabolic processes and catabolic repression involved in tolerance toward furfural

The RNA-seq data in response of *N. crassa* to furfural showed that “carbohydrate metabolic process” was the most significantly downregulated biological process GO term (GO:0005975, *P* value: 7.2e−5). To delineate the possible link between furfural and carbohydrate metabolism, we characterized the sensitivity of *N. crassa* to furfural in the presence of different carbon sources. When the media contained xylose or glycerol instead of sucrose, wild-type cells were significantly more tolerant (by approximately 15%) to 15 mM furfural. Tolerance was even more pronounced (about 30%) when *N. crassa* was cultured on carboxymethyl cellulose (CMC). When sucrose was replaced with other fermentable sugars such as glucose or fructose, no change in sensitivity to furfural was observed (inhibition with sucrose was 57% relative to control, glucose and fructose presented similar inhibition with difference in values of up to 5% of the standard error).

To understand if the effect of non-fermentable sugars in vivo might be related to furfural degradation within the intracellular fraction, we compared the ability of *N. crassa* cell extracts to reduce furfural. We monitored NAD(P)H depletion in cell-free extracts obtained from cultures grown overnight on either sucrose or CMC, with furfural as the substrate. The activity measured with both co-factors increased significantly when cultures were grown on CMC as compared to sucrose (by 215% for NADH and 165% for NADPH) (Fig. 3a). In contrast to cultures grown on sucrose, on CMC, the specific activity coupled with NADH was significantly higher than with NADPH (2540 and 1235 mU/mg protein, respectively). These results suggest that the increased tolerance of *N. crassa* to furfural in vivo may be due, to a major extent, to the elevation in the amount or activity of enzymes that can reduce furfural using NADH as a co-factor.

In filamentous ascomycete fungi, the utilization of alternate carbon sources is partially controlled by the zinc finger transcription factor CreA/CRE-1, which is a carbon catabolite repressor (CCR) protein [30, 34, 43–47]. Since our results suggested that non-fermentable sugars affected sensitivity to furfural in vivo and in vitro, we analyzed the ability of a *cre*-*1* deletion strain to grow on media containing sucrose supplemented with furfural. When grown on sucrose, Δ*cre*-*1* de-represses many genes, which mimics the behavior of wild-type on non-fermentable sugars [47]. Under these conditions, we found that Δ*cre*-*1* is 30% more tolerant to 15 mM of furfural in solid media, relative to wild-type cells. In a 96-well plate assay, the Δ*cre*-*1* mutant also exhibited a significantly shorter lag phase of 2.75 h following exposure to 60 mM furfural (a reduction of 2 h relative to the wild-type strain). The Δ*cre*-*1* mutant was also significantly more tolerant to 30 and 60 mM concentrations of the furan (30 and 51%, respectively) (Fig. 1). When CMC, known to suppress the growth arrest of the Δ*cre*-*1* mutant [47], was used as a sole carbon source, no significant difference in growth was evident as compared to the wild-type strain at 15 mM of furfural. These data suggest that CCR affects resistance to furfural in vivo and that this effect is mediated through CRE-1 regulatory pathways.

#### The *ahd*-*2* (NCU00378) gene is involved in tolerance toward furfural

In *S. cerevisiae,* furfural is detoxified by aldehyde dehydrogenases [39]. We, therefore, examined expression profiles for genes encoding predicted aldehyde dehydrogenases from our furfural RNA-seq dataset for those that displayed changes in their transcriptional pattern, and were either up- or downregulated when exposed to furfural. Five putative aldehyde dehydrogenase genes met these criteria: NCU03076, NCU03415, NCU09648, NCU00936 and NCU00378. The phenotypic consequences of exposing strains carrying a deletion of each of these predicted aldehyde dehydrogenase genes to 60 mM furfural were assessed: one mutant, ΔNCU00378, previously designated aldehyde dehydrogenase-2 (*ahd*-*2*) [48], was 43% more sensitive to 60 mM furfural than the wild type on solid media (Additional file 1: Table S5). This sensitivity of the ∆*ahd*-*2* mutant was observed only when cultured on solid medium. This strain did not exhibit an increase in the lag phase or inhibition of growth in 96-well plate assays (Fig. 1). *ahd*-*2* was previously reported to be involved in indole-3-pyruvic acid pathway-mediated biosynthesis of auxin in *N. crassa* [48]. Strains carrying deletions of the other identified aldehyde dehydrogenase genes (ΔNCU03076, ΔNCU03415, ΔNCU09648 or ΔNCU00936) did not show a growth phenotype that differed from the wild-type strain when exposed to furfural.

The AHD-2 protein is the closest homolog of the mitochondrial aldehyde dehydrogenase from yeast, Ald4 (*e* value 4e^−175^). This result was also supported by a bioinformatics-based prediction for mitochondrial localization as determined with pSORTII [49] and MitoProt II [50], and close structural homology (*P* value 4.5 × 10^−124^) to the human mitochondrial aldehyde dehydrogenase (PDB structure 1cw3 [51]). To confirm that deletion of *ahd*-*2* resulted in sensitivity to furfural, we targeted the wild-type *ahd*-*2* gene, placed under the regulation of the constitutive *tef*-*1* promotor, to the *his*-*3* locus in a ∆*ahd*-*2* strain. The P*tef1*-*ahd*-*2*; ∆*ahd*-*2* strain exhibited a significantly shorter lag phase after exposure to 60 mM of furfural, to 2.6 h (a reduction of 2.2 h relative to wild-type), and was significantly more tolerant (by 36%) than the wild-type to a similar concentration of the chemical (Fig. 1). These data suggest that *ahd*-*2* is involved in sensitivity to furfural.

### Attributes of furfural tolerance identified by a hypothesis-based approach

In an attempt to complement our initial RNA-seq-based approach to identify genes involved in the response to furfural, we employed a hypothesis-driven approach based on the functions of relevant genes predicted to be involved in the process of detoxification or tolerance to furans, particularly furfural or HMF, in *S. cerevisiae* [18, 19, 39, 52], *Escherichia coli* [53] and *Cupriavidus basilensis* HMF14 (a bacterium isolated from enriched HMF) [54] (Additional file 1: Table S4). The homologous *N. crassa* genes were identified by BlastP and the appropriate deletion strains were obtained from the FGSC (Additional file 1: Table S4) and analyzed for their ability to grow on media supplemented with semi-lethal concentrations of 20 mM furfural.

#### The *flr*-*1* (NCU05580) transporter is involved in tolerance to furfural

Out of the 25 mutants tested (Additional file 1: Table S4), only one strain exhibited a difference in tolerance to furfural. The deletion strain of the membrane transporter NCU05580, a member of the major facilitator superfamily, exhibited a ~ 38% slower growth rate compared to wild-type when cultured in the presence of 60 mM of furfural in solid media (Additional file 1: Table S5). This phenotypic attribute of ∆NCU05580 was observed only when the strain was cultured on solid medium. It did not show an increase in the lag phase or inhibition of growth in 96-well plate assays. NCU05580 is a homolog of *S. cerevisiae* FLR1 (fluconazole resistant-1), which has been linked with tolerance to fluconazole [55]. Furthermore, overexpression of *FLR1* in *S. cerevisiae* resulted in enhanced resistance to HMF [18]. Hence, we designated NCU05580 as *flr*-*1* (furfural resistance-1).

We hypothesized that constitutive expression of *flr*-*1* would improve tolerance of *N. crassa* to furfural and perhaps toward other inhibitory compounds. To examine that possibility, we constructed a plasmid with the *flr*-*1* gene under a constitutive promoter (*Pcpc*-*1*^*Δ2uorf*^). The P*cpc*-*1*::*flr*-*1* construct fully complemented the slower growth phenotype of the Δ*flr*-*1* mutant (Additional file 1: Figure S3A). The P*cpc*-*1::flr*-*1* strain was screened for sensitivity to furfural at different concentrations compared to wild type. The strain was more tolerant to 60 mM furfural in solid media compared to wild-type, where an improvement of ~ 60% in colony growth was evident (Additional file 1: Table S5). When subjected to the 96-well assay, the P*cpc*-*1::flr*-*1* strain showed a significantly shorter lag phase when grown in the presence of 60 mM furfural (1.63 h compared to 4.77 h in wild-type cells) and significantly more tolerance (an increase of 27% at 60 mM furfural) (Fig. 1). Since no significant difference was evident at lower concentrations of furfural, we monitored the expression level of *flr*-*1* by real-time PCR. After exposure of wild-type cells to 120 mM, but not to 30 or 60 mM furfural, the expression of *flr*-*1* was reduced by ~ 70%, as compared to the control (Additional file 1: Figure S3B). Based on the cumulative results obtained, we concluded that *flr*-*1* is relevant to tolerance toward furfural and that a strain that constitutively expresses *flr*-*1* can improve the growth on furfural.

### A random promotor-based tagged mutagenesis approach for identification of genes involved in tolerance to furfural

One of the strategies to generate furfural-tolerant *S. cerevisiae* mutants has been by overexpression of genes involved in the detoxification process [52]. Based on the data provided by constitutive expression of *ahd*-*2* and *flr*-*1*, reported in this study, we hypothesized that a similar approach could be a possible key to improve our understanding of molecular pathways of tolerance in *N. crassa*. To do so, we produced a random integrative library of *N. crassa*, based on the integration of pAZ11 that contained the constitutive *cpc*-*1*^Δ2uorf^ promotor, linked with a hygromycin selection marker. After transformation, the colonies were screened for the ability to grow on medium containing hygromycin overlaid with medium containing a high concentration (90 mM) of furfural. Among the transformants obtained, we further analyzed two strains, T112 and T361, which repeatedly exhibited tolerance to the furan in the initial 96-well plate screen.

#### NCU02488 is involved in tolerance to furfural

The transformant T112 showed a shorter lag phase when exposed to 15 and 30 mM furfural, to 0.36 and 1.22 h (a decrease of 0.6 and 0.94 h relative to wild-type cells, respectively), but not at a concentration of 60 mM (Fig. 1). To further characterize the effect of furfural on the early stages of fungal growth, we examined asexual spore germination rates in the presence of the compound. Addition of 30 mM of furfural to conidial suspensions of T112 did not change the germination rate, which was similar to the wild-type control after 4 and 6 h (Fig. 2). In contrast, hyphal growth of wild-type cells of *N. crassa* was more sensitive to the furan as compared to the T112 strain when analyzed in the 96-well plate assay (Fig. 1).

We previously found that the addition of HMF conferred changes in the intracellular and secreted protein profile in the white-rot fungus *Pleurotus ostreatus* [56]. Hence, we examined if the transformant T112 conferred any detectable changes in intracellular and secreted protein fractions of *N. crassa* after exposure to furfural. To test this, we extracted these fractions after addition of 60 and 120 mM furfural and compared the protein profiles to those of the wild type. Although intracellular protein profiles were similar, in the secreted fraction a change in a ~ 75 kDa band was observed, which accumulated in correlation with an increase in furfural concentration in wild-type cells. An opposite trend (a decrease in the band intensity at higher concentrations) was observed in the T112 strain (Additional file 1: Figure S4). The bands were sequenced by MS and identified as having high coverage of the same protein, encoded by NCU05137. This gene was previously identified as encoding a non-anchored cell wall protein-1 (*ncw*-*1*) [40]. NCU05137 is conserved in the genomes of a number of filamentous ascomycete fungi, and the deletion of *ncw*-*1* has been associated with increased endoglucanase and glucosidase activities [29]. When re-examining our RNA-seq data, we observed that *ncw*-*1* exhibited a fourfold decrease in expression after exposure to furfural (Additional file 1: Table S6). However, real-time PCR analysis results showed similar *ncw*-*1* expression levels with or without furfural in both wild-type and the T112 strains. While there was no significant difference between the germination rates of the ∆*ncw*-*1* mutant and the parental wild-type strain after 4 h in the presence of 30 mM of furfural, the ∆*ncw*-*1* mutant germinated 50% faster at the 6 h time point (Fig. 2). The ∆*ncw*-*1* exhibited similar tolerance to furfural as the wild type in solid media and in the 96-well assays (Fig. 1).

**Fig. 6:**
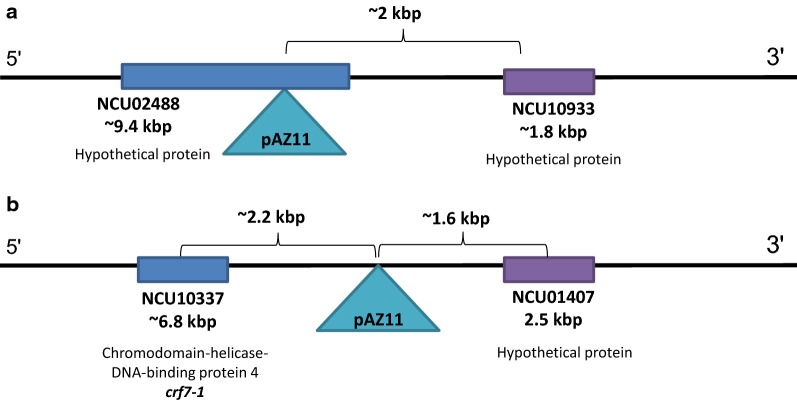
Schematic representation of plasmid insertion in transformants T112 (**a**) and T361 (**b**)

To identify the location of the pAZ11 insertion in transformant T112, the integration site was identified by genome sequencing, and localized in the first exon of NCU02488, a gene encoding for a predicted hypothetical protein (Fig. 6). We then analyzed the tolerance of the ∆NCU02488 strain to furfural. The deletion mutant had a significantly shorter lag phase after exposure to 30 mM and 60 mM furfural, to 0.09 and 2.34 h (reduction of 2.1 and 2.4 h relative to wild type, respectively) and was significantly more tolerant to 60 mM furfural by 28% (Fig. 1), as determined by the 96-well plate assay. Interestingly, the changes in NCW-1 protein levels in theΔNCU02488 strain were similar to T112 mutant (Additional file 1: Figure S4).

Transformant T361 exhibited an increase in tolerance to furfural. The transformant had a shorter lag phase of 2.33 h after exposure to 60 mM furfural (significant reduction of 2.1 h relative to wild type), but was not altered in relative growth rate as determined in the 96-well screen (Fig. 1). On solid medium, the tolerance of transformant T361 to 60 mM furfural was increased by 65% relative to wild-type (Additional file 1: Table S5). The mutant exhibited improved germination rate after addition of 30 mM of furfural, to 49% after 4 h (wild-type germination rate was 16%) and no inhibition was apparent after 6 h (wild-type germination rate was 47%) (Fig. 2).

The genome of transformant T361 was sequenced to locate the pAZ11 insert, which was integrated ~ 1.6 kbp upstream of NCU01407 (Fig. 6). The NCU01407 gene encodes a hypothetical protein, with a zinc finger C3H1-type profile (IPR000571). The ∆NCU01407 strain was only available as a heterokaryon and we were not able to produce a homokaryon with microconidia enrichment or sexual crossing. However, the heterokaryotic deletion strain had a significantly shorter lag phase after exposure to 15, 30 and 60 mM furfural, to 0.15, 0.56 and 1.63 h (a reduction of 0.8, 1.6 and 3.15 h relative to wild type, respectively) (Fig. 1), and was significantly (30%) more tolerant to 60 mM furfural (Fig. 1). Unlike in the case of the T112 strain, no noticeable changes were observed in the profiles of intracellular or secreted proteins.

## Discussion

The means for addressing the challenge of inhibitors produced during pretreatment of biomass were suggested to be a combination of introducing advanced detoxification methods, effective hydrolytic processes as well as molecular engineering of resistant phenotypes [57]. In this study, we focused on the ascomycete fungus *N. crassa* as a potential source of genes for yeast strain improvement. This fungus is able to grow on burned trees and is thus exposed to the inhibitory compound furfural in its natural habitat [25], even though the IC_50_ for furfural was similar in *N. crassa* and yeast [38]. To understand the molecular and metabolic basis and to identify new genes involved in tolerance toward furfural in *N. crassa,* we used a combination of approaches, including RNA-seq, hypothesis-driven reverse genetics and random promotor insertional mutagenesis.

We calibrated a high-throughput 96-well assay to measure the level of sensitivity of *N. crassa* to furfural, focusing on two main parameters; the delay in lag phase, and inhibition in relative growth, representing early colony inhibition and growth arrest, respectively. Furfural also inhibited fungal conidial germination. The screens of different mutants in this study showed that these three parameters, germination, lag phase and growth inhibition, do not overlap and different genes can be involved in each process. Among the genes we identified as relevant to furfural tolerance, *ncw*-*1* has an effect only during germination, *ahd*-*2* and *flr*-*1* improved tolerance during hyphal growth, while *cre*-*1* and NCU02488 were involved in both phases.

RNA-seq analyses revealed that a total of 936 genes were differentially expressed after exposure to furfural. This transcriptional response was similar to other organisms, such as *S. cerevisiae* [19, 58, 59] and the ethanol producing Gram-negative bacterium *Zymomonas mobili*s [60]. Comparing the response between furfural and HMF (Additional file 1: Figure S2) showed that *N. crassa* responded differently to each furan, and only a small number of the altered genes overlapped. Furfural affected the expression of 3.5-fold more genes than HMF, in either an upregulated or downregulated manner. These data suggest a more global response of *N. crassa* toward furfural, perhaps since it is more specialized in metabolizing a compound that can be found in its natural habitat [25].

The RNA-seq results showed that the GO term of “carbohydrate metabolic process” was affected by furfural. Likewise, comparative proteomic analysis of a new furfural-adapted *Pichia stipitis* strain (Y7-1) found that the most differentially expressed proteins are involved in carbohydrate metabolism [61]. *Lactobacillus brevis* S3F4 showed strong resistance to fermentation inhibitors such as furfural and ferulic acid under CCR conditions when grown on xylose [62]. Increased tolerance to furfural was observed in *N. crassa* when CMC was used as carbon source, coupled with improved in vitro furfural degradation utilizing NADH as a co-factor. A similar response was observed in a *cre*-*1* mutant, which bears a deletion in a key transcription factor important for regulating CCR [30, 34, 43–47]*. N. crassa* has significant cellulolytic activity [29, 43, 63] and our data indicate that under these conditions, it is more tolerant to furfural.

Furfural was shown to inhibit important metabolic enzymes including aldehyde dehydrogenase [39, 64]. Using a reverse genetics approach, we found that a deletion mutant of an aldehyde dehydrogenase gene (*ahd*-*2*) conferred sensitivity to furfural. *ahd*-*2* is the closest homolog of Ald4 in *S. cerevisiae*, which is able to reduce HMF and furfural utilizing NADH as a co-factor [39], and was induced in the transcriptome immediately after exposure to HMF [19]. Constitutive expression of *ahd*-*2* conferred improved tolerance to furfural, while the deletion of Ald4 in *S. cerevisiae* did not affect the growth with either furfural or HMF, and the overexpression strain failed to grow in the presence of HMF [39]. The involvement of aldehyde dehydrogenases in tolerance toward furfural seems to be phylogenetically conserved, as they are important attributes in tolerance to furans both in yeast and *N. crassa*. AHD-2, unlike Ald4 from *S. cerevisiae*, is not redundant in its role in furfural tolerance by other aldehyde dehydrogenases. This result emphasizes the conserved, but not identical mode of resistance, between these two fungi.

A hypothesis-based approach was used to screen potential genes that were previously reported to have an effect on tolerance to furfural. A strain in which *flr*-*1* (NCU05580) had been deleted exhibited slower growth coupled with increased sensitivity to furfural. FLR-1 harbors major a facilitator superfamily (MFS) domain (IPR020846), whose members function as transporters. Constitutive expression of *flr*-*1* resulted in an increased tolerance to high concentrations of furfural. As *flr*-*1* expression is downregulated at high concentrations of furfural, it is conceivable that P*cpc1*::*flr*-*1* “masks” that effect conferring improved tolerance. FLR-1 is the closest homolog to Flr1 in *S. cerevisiae*, whose overexpression resulted in enhanced resistance to the phenolic inhibitor coniferyl aldehyde and to HMF [18]. *flr*-*1* is also a homolog of the Tpo1p, a transporter of polyamines. However, unlike *flr*-*1*, *TPO1* expression was induced by HMF during the lag phase in *S. cerevisiae* [19]. Overall, we propose that further analysis of *flr*-*1* can improve our understanding of the fungal cellular response to furfural. Furthermore, as the *FLR1* and *TOP1* homologs are linked to tolerance to HMF, but not to furfural in *S. cerevisiae*, *N. crassa flr*-*1* could well be a valid candidate for heterologous expression in yeast.

The fact that constitutive expression of *ahd*-*2* and *flr*-*1* conferred improved tolerance to furfural encouraged us to further exploit the possibility of using promotor-tagged random mutagenesis to generate strains with improved tolerance to this furan. The transformant T112 had a significantly shorter lag phase, but increased inhibition in relative growth, after exposure to furfural. The insertion was within the NCU02488, which encodes a small hypothetical protein of 188 aa, which is specific to *N. crassa* and closely related species, such as *N. tetrasperma* and *Sordaria macrospora*. The ∆NCU02488 mutant also exhibited a decreased lag phase but, unlike T112, was more tolerant in relative growth to furfural. The transformant T112 exhibited a decrease in NCW-1 accumulation in the secretome; the ∆*ncw*-*1* strain was previously shown to have increased endoglucanase and glucosidase activities [29]. Via RNA-seq analyses, we showed that these same activities decreased by fourfold after exposure to furfural, suggesting yet another link between CCR and furfural tolerance in *N. crassa*. A *ncw*-*1* deletion strain had similar growth sensitivity to furfural as the wild-type strain, but improved germination after exposure to the compound. This result suggests that improved tolerance toward furfural during germination in T112 may be partially mediated by NCW-1. Similar to T112, transformant T361 had a significantly shorter lag phase after exposure to furfural. The plasmid insertion was upstream of NCU01407, which encodes a hypothetical protein of 495 aa. A ∆NCU01407 mutant conferred increased tolerance to furfural and decreased lag phase. The gene harbors an InterPro domain of zinc finger C3H1-type profile (IPR000571). In *N. discreta*, the NCU01407 ortholog (NEUDI_17858) has been designated as an SWI-SNF chromatin-remodeling complex protein, suggesting that tolerance to furfural may involve the chromatin-remodeling complex.

This study presents different and complementary approaches to identify novel genes linked to furfural resistance in *N. crassa*, during which we managed to reveal the involvement of six genes. Among them only two, *ahd*-*2* and *ncw*-*1*, were differentially expressed after exposure to furfural (Additional file 1: Table S6). Four of the genes were novel targets to furfural tolerance, and only *flr*-*1* and *ahd*-*2* have homologs in *S. cerevisiae,* previously reported to be involved in resistance to HMF, suggesting a different mode of tolerance to furans in these two fungi. Screening deletion mutants of genes that were differentially affected by furfural, revealed that, similar to yeast [39], *N. crassa* has a redundancy in genes relevant to tolerance mechanisms. Using both hypothesis-driven and random mutagenesis approaches, we were able to identify additional attributes and to improve our understanding regarding the genetic mechanism of tolerance.

## Conclusions

The results presented here demonstrate that furfural inhibits *N. crassa* in two modes; it delayed conidial germination and conferred mycelium growth arrest. The transcriptional response to furfural was similar to yeast, with a more global reaction toward furfural than to HMF. The RNA-seq results led us to link the response to carbohydrate metabolism and *cre*-*1*, as well as the aldehyde dehydrogenase *ahd*-*2*. In the hypothesis-based screen in which known genes were investigated, we identified the *flr*-*1* transporter, and in random promotor mutagenesis we identified NCU02488 and NCU01407. We propose that these newly identified genes can be heterologously expressed in yeast to improve tolerance to furfural. We also propose that a similar research outline, as presented in this study, could be utilized to identify novel resistance attributes not only for furans but to other toxic chemicals and in other organisms.

## Materials and methods

### Fungal growth conditions

General procedures and media used in the handling of *N. crassa* have been previously described [21] or are available through the FGSC (http://www.fgsc.net/Neurospora/NeurosporaProtocolGuide.htm). Conidial germination assays was performed as previously described [65].

*Neurospora crassa* strains used in this study are specified in Additional file 1: Table S7. Vogel’s minimal medium [21] was used in this study, supplemented with 1.5% (w/v) sucrose (VgS) or other carbon sources if mentioned otherwise. When required, 1.5% agar was added. For restricting *N. crassa* growth, Vogel’s minimal medium was supplemented with FGS (2% l-sorbose, 0.05% glucose and 0.05% fructose). When required, the medium was supplemented with 100 µg/ml histidine (Sigma-Aldrich) and/or 10 µg/ml hygromycin B (Calbiochem, Riverside, CA, USA). Throughout the study, we used different concentrations of furfural (2-furaldehyde 99%, Sigma-Aldrich) and HMF (5-hydroxymethyl furfural ≥ 99%, Sigma-Aldrich).

### Growth of *N. crassa* in 96-well plate format

*Neurospora crassa* was grown in 96-well plates as described by Lopez-Moya et al. [66]. Briefly, the growth of the different strains was followed using 96-well plates (TC microwell 96F, Thermo Fisher Scientific, Denmark). Conidia (2 × 10^6^ spores/ml) were inoculated into 200 µl of liquid VgS media with or without furfural, per well. Plates were incubated stationary at 34 °C in a Synergy HTX Multi-Mode Microplate Reader (BioTek, Winooski, USA); absorbance (490 nm) was measured every 15 min for 20–21 h. The blank controls contained media with the relevant concentrations of furfural without conidia. For calculations, we subtracted the blank controls and the absorbance at time zero from the relevant experimental samples. Experiments were repeated at least six times, in triplicate.

### Enzymatic reaction for degradation of furfural in vitro

Furfural depletion coupled with NAD(P)H was measured according to Liu et al. [39]. *N. crassa* strains were grown for 16 h in 100 ml Erlenmeyer flasks containing 20 ml of medium. Mycelial samples were disrupted using a Bead Beater (BioSpec Products, Inc., Bartlesville, OK, USA) in 500 μl of 100 mM potassium phosphate buffer (pH = 7). The homogenates were centrifuged for 1 min at 4000×*g* at 4 °C. The protein concentration of the clear lysate was determined using the BioRad protein assay kit (BioRad, Hercules, CA, USA). A 30 μl sample of lysate was used for each reaction. After the NAD(P)H was added to the protein lysate, the activity was assayed spectrophotometrically at 340 nm (*ε* 340 = 6220 M^−1^ cm^−1^) with 10 mM furfural in 100 mM of potassium phosphate buffer. The assay was conducted in a total volume of 200 μl, in microtiter plates, at 30 °C, and changes in absorption were monitored for 15 min, using the Microplate Reader. The data from controls without furfural, as well as samples that were treated at 95 °C for 5 min were subtracted from the treatments. An enzyme unit was defined as the amount of enzyme depleting 1 μmol of NAD(P)H per minute per mg protein. The experiments were performed in three biological replicates (each treatment was composed of three culture flasks and the experiment was repeated on three different dates).

### RNA-seq data analysis

Strain FGSC 2489 was grown on VgS slants for 7 days. Conidia were inoculated into 9 × 100 ml fresh VgS for each concentration of furan to a final concentration of 1 × 10^6^ per ml and grown at 30 °C for 16 h, at constant light and shaking (200 rpm). The mycelia were washed 2 times with 100 ml minimal medium lacking sucrose and were re-suspended into fresh VgS containing 10 mM furfural or HMF for 4 h, at 30 °C, in constant light and shaking (200 rpm). DMSO (1.0%, v/v) was used as a solvent control. Four hours after inoculation, three samples were each filtered through a Whatman #1 filter and dried to completion at 75 °C overnight for biomass measurements. Mycelial mass was reduced to 35% (w/w) for furfural and 30% (w/w) for HMF. An additional three replicates of mycelia were filtered through Whatman paper and snap frozen in liquid nitrogen for RNA extraction.

Total RNA was isolated from the frozen tissue by the Trizol–Phenol–Chloroform method (Thermo Fisher Scientific). The extract was digested with TURBO DNAse (Life Technologies). The poly(A) + mRNA was purified from 10 µg total RNA using Dynabeads oligo(dT) magnetic beads (Life Technologies). The mRNA was chemically fragmented using the Ambion RNA fragmentation kit (Life Technologies). The Illumina TruSeq kit was employed to generate the cDNA libraries with indexing adapters essentially following the manufacturer’s protocol. The cDNA was size fractioned by gel electrophoresis to 200 bp and then amplified by PCR. The cDNA libraries were sequenced as 50 bp single-end reads on an Illumina HiSeq 2000 platform.

Low quality reads with base calls having a *Q* value < 20 were removed using the FASTX toolkit (http://hannonlab.cshl.edu/fastx_toolkit/download.html) and the filtered reads were mapped to predicted transcripts from the *N. crassa* OR74A genome v12 using Tophat v2.04 [67]. Expression values were normalized as FPKM with cufflinks v2.02 [68] using compatible hits as a normalizing option corrected for fragment and multiple hit biases to the genome. Individual genes that had a differential expression log2 > 1.0 and an adjusted *P* value < 0.01 after a multiple testing correction using Benjamini–Hochberg–procedure were considered significantly expressed genes using the cuffdiff component of cufflinks v2.02 [68]. Significantly expressed genes were used for hierarchical clustering with Cluster 3.0 [69]. Genes were log transformed, normalized across all conditions and centered on a geometric mean on a per gene basis. Clustering was performed as an average linkage with an uncentered correlation as the similarity metric. Whole RNA-seq libraries were clustered by Euclidean distance using a variance-stabilizing transformation module in DEseq v1.14. SRA accession: PRJNA541531.

Functional analysis of differentially expressed genes was based on Gene Ontology (GO) groups as annotated by the Blast2GO v2.8 package [70]. A BLASTP of the *N. crassa* OR74A protein database to the non-redundant protein database https://blast.ncbi.nlm.nih.gov/ was generated to create a reference annotation GO term file for FGSC2489. Enrichment tests for GO terms were determined by a one-tailed Fishers exact test with a multiple testing correction cutoff of 0.05.

### Gene expression analyses

The RNA samples were purified with the RNeasy Plant Mini Kit (Qiagen, Hilden, Germany) and then treated with the DNA-free kit (Ambion). 1 μg of purified RNA was used for cDNA synthesis utilizing super-script II RNase H reverse transcriptase (Invitrogen, Carlsbad, CA, USA). Relative quantification of the transcript abundance was performed using an ABI StepOnePlus Real-Time PCR sequence detection system and software (Applied Biosystems). The PCR contained 20 ng of total cDNA and 300 nM oligonucleotide primers (Additional file 1: Table S8), β-tubulin as endogenous control [71]. Amplification data were compared on the basis of the of ΔCT method and presented as 2^−ΔCT^ or ΔΔCT method and presented as 2^−ΔΔCT^.

### Construction of Ptef1-ahd-2-gfp

A PCR fragment was amplified from FGSC 2489 genomic DNA with the primers NCU00378_XbaI_F and NCU00378_PacI_R (Additional file 1: Table S8) using Phusion DNA polymerase (NEB # M0530). The final product was subjected to PCR purification with Qiaquick PCR kit (Catalog # 28104) and cloned into the pCR^®^ vector (Invitrogen # 44-0302) according to the manufacturer’s protocol. The NCU00378 insert was excised from the vector with *Xba*I and *Pac*I and cloned into a modified pMF272 vector cut with *Xba*I/*Pac*I in frame with an eGFP reporter ORF and flanked by *tef*-*I* promoter and *ccg*-*1* terminator sequences from pMF272 [72]. The deletion strains for NCU00378 (FGSC 12919) were crossed with FGSC 6103 to create an NCU00378 deletion in a *his3*^−^ background. Progeny were genotyped by Phire Plant Direct PCR (Thermo Scientific # F-130WH) on conidia with the primers Hphp and NCU00378_3r (Additional file 1: Table S8).

Transformation was performed by electroporation according to a protocol available at the FGSC or as modified by Ziv and Yarden [73]. Conidia from the selected transformants were genotyped by PCR for the hygromycin cassette and also for the NCU00378-GFP construct with the primers NCU00378_F and pMF272_Rev (Additional file 1: Table S8).

### Construction of *Pcpc*-*1*^*Δ2uorf*^-*flr*-*1*

To construct the *flr*-*1* gene with constitutive expression under *Pcpc*-*1*^*Δ2uorf,*^ the promotor was amplified from pMP6 [74] with primers pMP6-181F and pMP6-11742R. The NCU05580 gene along with its 3′ flank was amplified from genomic DNA of *N. crassa* with NCU05580-2340R and NCU05580-25F linker (with linker to *Pcpc*-*1*^*Δ2uorf*^ from pMP6) (Additional file 1: Table S8). The 5′ flank of *flr*-*1* was amplified from genomic DNA with NCU05580-637F and NCU05580-54R linker (with linker to -*Pcpc*-*1*^*Δ2uorf*^*)* (Additional file 1: Table S8). The three segments were joined together using the Phusion™ High-Fidelity PCR Master Mix (Thermo Scientific, USA). The amplified construct was cloned into pDRIVE and designated pDF2. pDF2 was digested with *Xba*I and *Sma*I and the resulting segment was then co-transformed with pCZ67 (containing the *his*-*3* gene for selection) into ∆*ku80*; ∆*his3*^−^ to replace the existing copy of NCU05580. For complementation, pDF2 was transformed into the ∆*flr*-*1*; ∆*his3*^−^ strain. The selection of the transformants was based on their ability to grow without histidine, followed by verification of the insertion by PCR.

### Promotor-based tagged mutagenesis

pAZ11 contains P*cpc*-1^Δ2uorf^ from pMP6, along with the hygromycin phosphotransferase gene, from pCSN44. pAZ11 was linearized with *Apa*I and the random mutagenesis procedure was performed using electroporation, into the wild-type strain (FGSC #987). Conidia subjected to transformation were then plated on Vogel + FGS amended with hygromycin. After the colonies began to form the plates were overlaid with Vogel + FGS supplemented with 90 mM furfural. Emerging colonies were further analyzed for their tolerance to furfural.

### Secreted proteins profiles

Twenty milliliters of conidia (2 * 10^5^ spores/ml) were used to inoculate 100 ml Erlenmeyer flasks containing VgS and grown for 17 h, 150 rpm at 34 °C. The relevant cultures were than supplemented with 60 or 130 mM of furfural and were grown for additional 3.5 h. For extracellular protein analyses, culture fluids were filtered through Whatman No. 1 filter paper followed by an additional filtration step using 0.45-μm mixed cellulose ester filter paper (Whatman, Buckinghamshire, UK). Each sample was then concentrated using an Amicon^®^ Ultra 0.5/Ultra 15 (Millipore, Billerica, MA, USA) system and supplemented with complete (Roche Applied Science, Mannheim, Germany), after the concentration process. The protein concentration was determined using the BioRad protein assay kit (BioRad, Hercules, CA, USA). The proteins were separated and visualized with Coomassie R-250 (0.125%). Each sample was subsequently analyzed by HPLC/mass spectrometry/mass spectrometry (LC–MS/MS) in an Orbitrap (Thermo Scientific, Waltham, MA, USA) mass spectrometer and identified by Sequest 3.31 software against the genome of *N. crassa* OR-74A in NCBI. Protein identification was performed by the Smoler Proteomics Center of The Israel Institute of Technology (Technion).

### Sequencing of transformants T112 and T361

#### Library construction and sequencing

DNA-Seq was performed as previously described [75] with the following modifications: 300–600 ng of gDNA was sheared using the Covaris E220X sonicator (Covaris, Inc., Woburn, MA, USA). End repair was performed in 80ul reaction at 20 °C for 30 min. Following Agencourt AmPURE XP beads cleanup (Beckman Coulter, Inc., Indianapolis, IN, USA) in a ratio of 0.75× Beads/DNA volume, adenine bases were added to both 3′ ends followed by adapter ligation in a final concentration of 0.125 μM. An SPRI bead cleanup in a ratio of 0.75× beads/DNA volume was performed, followed by 8 PCR cycles using 2× KAPA HiFi ready mix (Kappa Biosystems, Inc. Wilmington, MA, USA) in a total volume of 25 μl with the following program: 2 min at 98 °C, 8 cycles of 20 s at 98 °C, 30 s at 55 °C, 60 s at 72 °C following by 72 °C at 10 min.

Libraries were evaluated by Qubit and TapeStation. Sequencing libraries were constructed with barcodes to allow multiplexing of nine samples in one lane of Illumina HiSeq 2500 V4 instrument, using paired end 125 bp protocol. The output was ~ 23 million reads per sample. Sequencing depth was homogenous across samples. Fastq files for each sample were generated by the usage of Illumina bcl2fastq v2.17.1.14 software.

#### Analysis of sequence data

A reference genome which is based on *N. crassa* OR74 genome (FungiDB [76], release 30) and the given plasmid, pAZ11, as an additional contig was prepared. Speedseq framework [77] (version 0.0.3a) was used for the genome analysis to detect structural variants. The framework includes the following steps: (i) align—reads for each sample were mapped independently using BWA-MEM [78] to the reference genome. Duplicate reads were marked by SAMBLASTER [79]. 97% uniquely mapping rates was observed. The mapped reads yielded a mean coverage depth of 20× per sample and above 99% of the genome is covered with more than 25×. (ii) sv—run LUMPY [80] on all samples. This pipeline infers genomic variations: single nucleotide variants (SNPs), indels, and structural variants (SVs).

#### Bioinformatics analysis

To detect events that are related to the plasmid insertions, we extracted variants which were involved with the plasmid contig. The evidence for the insertion is supported by (1) detected breakpoint within the reads (by split reads) and (2) discordant read pairs for which one of the pairs maps to the plasmid. Mind The Gap [81] was used to validate the plasmid events by assembly of insertion for detected breakpoints.

DNA-seq and relevant bioinformatics of the transformants were carried out at the Nancy and Stephen Grand Israel National Center for Personalized Medicine, Rehovot, Israel.

## Supplementary information

**Additional file 1: Figure S1.** Inhibitory effect of furfural on *N. crassa* wild-type *in* solid media. **Figure S2.** GO terms associated with biological processes that are up regulated in *N. crassa,* upon exposure to HMF. **Figure S3.** Complementation of ΔNCU05580 (A) and reduced expression of NCU05580 after exposure to 120 mM of furfural. **Figure S4.** Profiles of secreted proteins obtained from wild-type, T112 and ∆NCU02488 cultures grown in the presence of furfural. **Table S2.** Notable genes whose expression levels were increased following exposure to furfural. **Table S3.** Summary of genes that had the highest up- or downregulation in response to furfural or HMF and were screened for tolerance to the furans. **Table S4.** Summary of genes whose deletion strains were analyzed in a hypothesis-driven approach. **Table S5.** Inhibition in relative growth of different mutant strains in the presence of 60 mM of furfural, on solid media. The presented results were normalized to wild-type. The values and standard errors represent the average of at least three biological replicates. **Table S6.** Summary of genes identified in this study that are involved in tolerance to furfural. **Table S7.** Strains of *N. crassa* used in this study. **Table S8.** Oligonucleotides used in this study.

**Additional file 2: Table S1.** Differentially expressed genes by RNA-seq in *N. crassa*, after exposure to 10 mM furfural or 10 mM HMF

**Additional file 3: Table S9**. Gene Ontology terms for biological processes with significantly over expressed genes upon exposure to furfural.
