# Supplementary material for: Identification and manipulation of Neurospora crassa genes involved in sensitivity to furfural
Source: Biotechnol Biofuels. 2019 Sep 4;12:210. doi: 10.1186/s13068-019-1550-4 (PMC6724289; doi:10.1186/s13068-019-1550-4)
Supplement: Supplementary file 1 — Additional file 1: Figure S1. Inhibitory effect of furfural on N. crassa wild-type in solid media. Figure S2. GO terms associated with biological processes that are up regulated in N. crassa, upon exposure to HMF. Figure S3. Complementation of ΔNCU05580 (A) and reduced expression of NCU05580 after exposure to 120 mM of furfural. Figure S4. Profiles of secreted proteins obtained from wild-type, T112 and ∆NCU02488 cultures grown in the presence of furfural. Table S2. Notable genes whose expression levels were increased following exposure to furfural. Table S3. Summary of genes that had the highest up- or downregulation in response to furfural or HMF and were screened for tolerance to the furans. Table S4. Summary of genes whose deletion strains were analyzed in a hypothesis-driven approach. Table S5. Inhibition in relative growth of different mutant strains in the presence of 60 mM of furfural, on solid media. The presented results were normalized to wild-type. The values and standard errors represent the average of at least three biological replicates. Table S6. Summary of genes identified in this study that are involved in tolerance to furfural. Table S7. Strains of N. crassa used in this study. Table S8. Oligonucleotides used in this study. [file 13068_2019_1550_MOESM1_ESM.docx]

**Additional file 1:**

***Identification and manipulation of Neurospora crassa genes involved in sensitivity to furfural***

Daria Feldman^a#^, David J. Kowbel^b#^, Adi Cohen^a^, N. Louise Glass^b,c^, Yitzhak Hadar^a^ and Oded Yarden^a*^

^a^Department of Plant Pathology and Microbiology, The R.H. Smith Faculty Agriculture, Food and Environment, The Hebrew University of Jerusalem, Rehovot 7600001, Israel; ^b^Department of Plant and Microbial Biology, University of California, Berkeley, Berkeley, CA 94720, USA; ^c^Environmental Genomics and Systems Biology Division, The Lawrence Berkeley National Laboratory, 1 Cyclotron Road, Berkeley CA 94720.

# Equal contributors

*Address correspondence to Oded Yarden, oded.yarden@mail.huji.ac.il

**
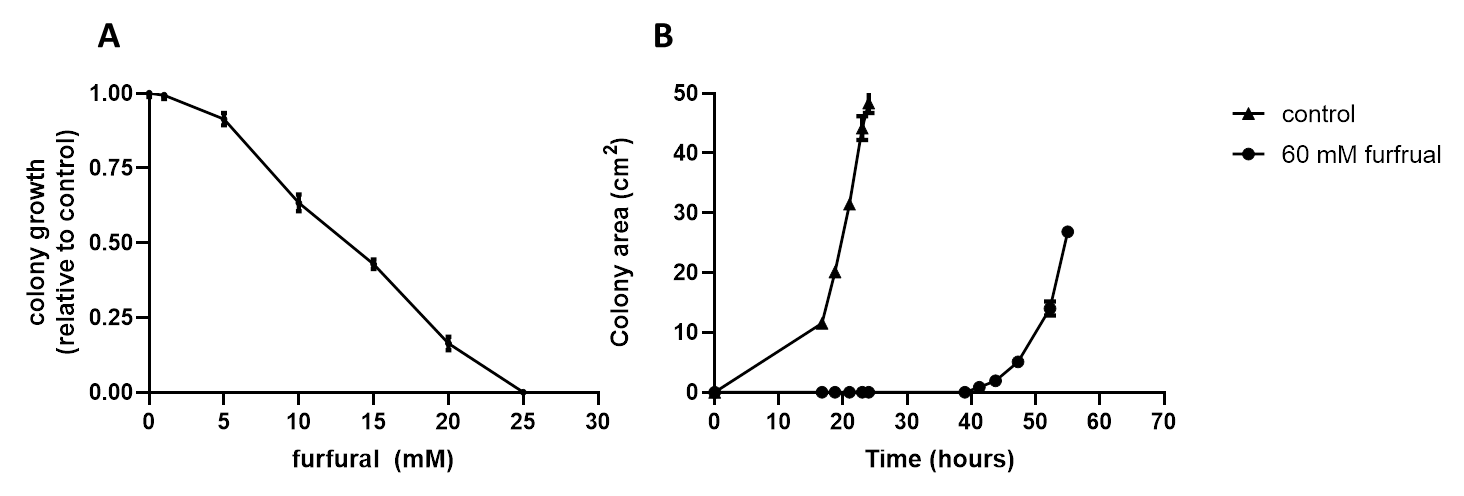
**

**Figure S1**: Inhibitory effect of furfural on *N. crassa* wild-type *in* solid media

(**A**) The linear growth was measured following exposure of wild-type conidia to different concentrations of furfural. Inhibition was measured relative to control. Bars indicate standard errors.

(**B**) Colony growth of wild-type supplemented with 60 mM of furfural. The colony area of wild-type *N. crassa* grown on solid VgS with or without 60 mM furfural measured over time.

The values represent the average of three biological replicates.

**
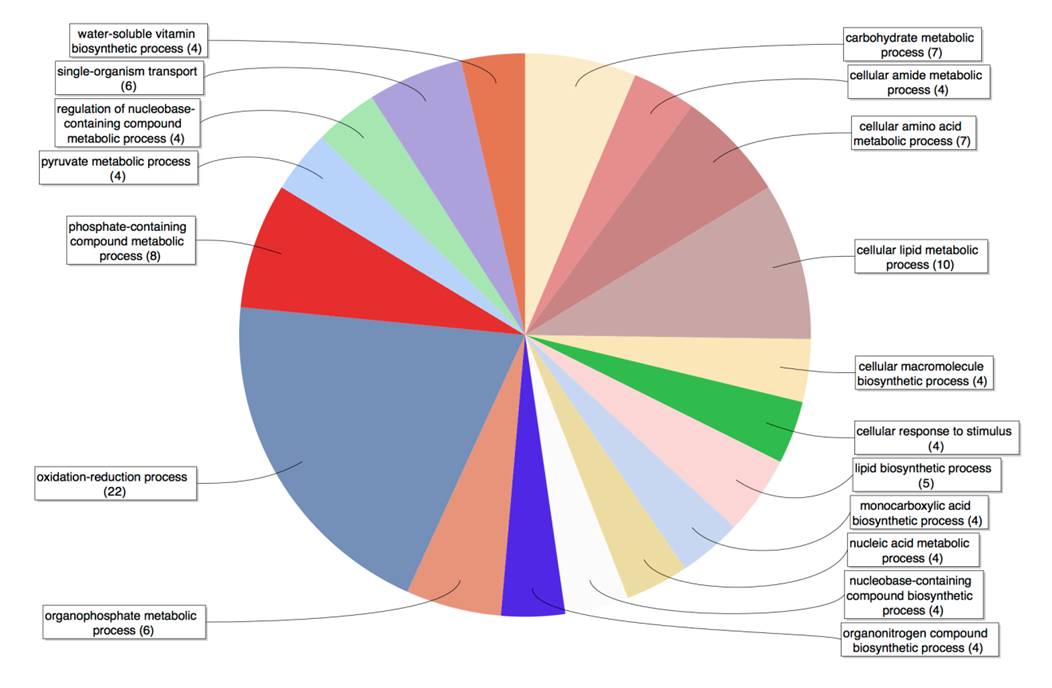
**

**Figure S2:** GO-terms associated with biological processes that are up regulated in *N. crassa,* upon exposure to HMF.

GO-terms were associated with all protein coding genes using the program Blast2Go v 2.8 [69] and a multilevel pie chart generated from genes that are up regulated with a log2 value > 1.0 and p-adjusted value < 0.01 and a node score > 3.0. Only terminal nodes are presented in the pie chart with the node score for each biological process in parentheses.

**
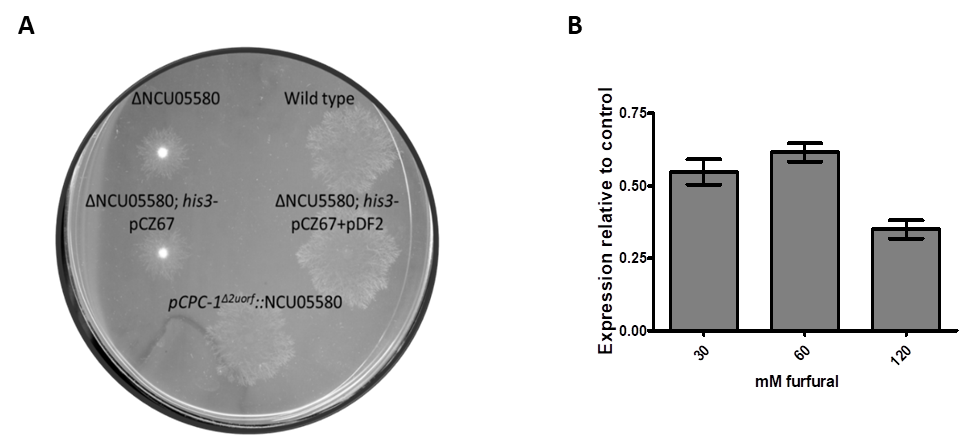
**

**Figure S3:** Complementation of ΔNCU05580 (**A**) and reduced expression of NCU05580 after exposure to 120 mM of furfural.

(**A**) Cotransformation of ΔNCU0558 with pDF2 with pCZ67 (harbouring a *his3* gene) completely restored the wild type phenotype.

(**B**) Expression levels of NCU05580 were monitored by real-time RT-PCR. *N. crassa* was grown in liquid culture for 16 hr, after different addition of different concentrations of furfural (30, 60 and 120mM). The RNA was extracted 4 hr after exposure. The expression levels were calculated relative to β-tubulin, as the endogenous control, and represent the expression relative to control without furfural addition. Bars indicate standard errors. The values represent the average of three biological replicates.

**
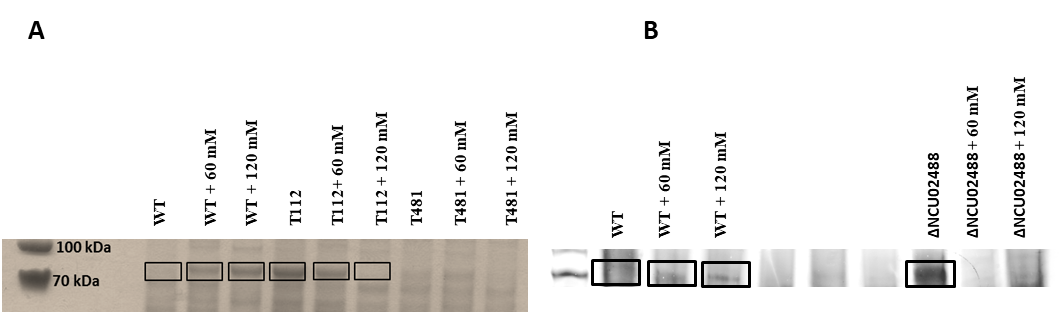
**

**Figure S4**: Profiles of secreted proteins obtained from wild-type, T112 and ∆NCU02488 cultures grown in the presence of furfural.

1. Wild-type, strain T112 and T481 (control strain with random integration of pAZ11).
2. Wild-type and ∆NCU02488 (separated by the unmarked lanes).
3. and (B) are from two separate experiments. In both cases the cultures were grown for 17 hr and then supplemented with 60 or 120 mM of furfural. The secreted proteins were concentrated 3.5 hr after exposure to the furan. NCU05137 (NCW-1) was identified by MS and is marked on the gel. The results were verified in 3 independent experiments.

**Table S2:** Notable genes whose expression levels were increased following exposure to furfural

| **Gene** | **Annotation** | **Mechanism** | **log2** | **Fold change** |
| --- | --- | --- | --- | --- |
| NCU07953 | alternative oxidase-1, *aod-1* | Suggesting the occurrence of mitochondrial damage, possibly due to reactive oxygen species (ROS) production | 5.17 | 35.91 |
| NCU09864 | 2-oxoisovalerate dehydrogenase alpha subunit | Branched-chain amino acid synthesis | 2.94 | 7.66 |
| NCU03913 | 2-oxoisovalerate dehydrogenase beta subunit | Branched-chain amino acid synthesis | 3.50 | 11.36 |
| NCU02704 | branched-chain alpha-keto acid dehydrogenase E2 component | Branched-chain amino acid synthesis | 5.00 | 32.09 |

**Table S3:** Summary of genes that had the highest up- or down-regulation in response to furfural or HMF and were screened for tolerance to the furans.

| **Gene** | **Annotation** | **FGSC strain** | **furfural log2(foldchange)** | **HMF log2(foldchange)** |
| --- | --- | --- | --- | --- |
| NCU04697 | cyanide hydratase | FGSC #11824 | 7.56 | 4.49 |
| NCU00732 | trichothecene C-15 hydroxylase/ cytochrome P450 | FGSC #18706 | 5.72 | 5.88 |
| NCU07953 | alternative oxidase-1, aod-1 | FGSC #18947 | 5.17 | NS |
| NCU04591 | pentachlorophenol monooxygenase | FGSC #16793 | 4.62 | NS |
| NCU05045 | MFS monocarboxylate transporter | FGSC #13435 | 4.61 | NS |
| NCU08561 | succinate/fumarate mitochondrial transporter | FGSC #20330 | 3.68 | NS |
| NCU08096 | hypothetical protein | FGSC #20515 | 3.66 | NS |
| NCU08907 | BYS1 domain-containing protein, ccg-13 | FGSC #18423 | -8.72 | NS |
| NCU08720 | hypothetical protein | FGSC #20454 | -6.90 | NS |
| NCU08457 | easily wettable, eas | FGSC #13319 | -6.36 | -5.44 |
| NCU06912 | hypothetical protein | FGSC #12948 | -6.03 | NS |
| NCU00399 | cell wall protein PhiA | FGSC #17647 | -5.99 | NS |
| NCU04478 | phospholipase D active site-containing protein | FGSC #18769 | NS | 4.14 |
| NCU09141 | pyridoxal reductase | FGSC #14567 | NS | 3.78 |
| NCU09648 | aldehyde dehydrogenase | FGSC #14354 | NS | 3.38 |
| NCU09210 | dyp-type peroxidase | FGSC #14417 | -2.02 | -5.46 |
| NCU09209 | galactose oxidase | FGSC #14415 | -1.98 | -3.60 |
| NCU05629 | hypothetical protein | FGSC #17683 | -1.08 | -3.38 |

**Table S4:** Summary of genes whose deletion strains were analyzed in a hypothesis-driven approach.

| **Gene** | **homolog gene** | **Annotation** | **FGSC strain** | **organism** | **reference** |
| --- | --- | --- | --- | --- | --- |
| NCU03350 | >gi\|291619937\|gb\|ADE20401.1\| furoyl-CoA dehydrogenase 2Fe-2S iron sulfur subunit | xanthine dehydrogenase | FGSC #13065 | *C. basilensis* HMF14 | [53] |
| NCU08935 | >gi\|291619938\|gb\|ADE20402.1\| furoyl-CoA syntethase | peroxisomal-coenzyme A synthetase | FGSC #18426 | *C. basilensis* HMF14 | [53] |
| NCU09798 | gi\|291619945\|gb\|ADE20408.1\| HMF/furfural oxidoreductase | aryl-alcohol dehydrogenase | FGSC #18587 | *C. basilensis* HMF14 | [53] |
| NCU04078 | >gi\|301015215\|gb\|ADK47404.1\| YqhD | NAD-dependent methanol dehydrogenase | FGSC #10753 | *E. coli* | [52] |
| NCU04510 | >gi\|301015216\|gb\|ADK47405.1\| DkgA | aldose reductase | FGSC #16700 | *E. coli* | [52] |
| NCU09390 | >gi\|388478470\|ref\|YP_490662.1\| oxidoredutase, sulfate metabolism protein | tetrahydroxynaphthalene reductase | FGSC #17987 | *E. coli* | [52] |
| NCU09519 | >gi\|301015216\|gb\|ADK47405.1\| DkgA | 2,5-diketo-D-gluconic acid reductase A | FGSC #21151 | *E. coli* | [52] |
| NCU05580 | >gi\|gb\| DAA07129.1\| Flr1p | membrane transporter | FGSC #13741 | *S. cerevisiae* | [18] |
| NCU08384 | >gi\|256273232\|gb\|EEU08178.1\| Gre3p | xylose reductase | FGSC #20307 | *S. cerevisiae* | [38] |
| NCU05591 | >gi\|1420383\|emb\|CAA99359.1\| PDR5  >gi\|927337\|gb\|AAB64846.1\| Pdr15p | ABC transporter CDR4 | FGSC #11238 | *S. cerevisiae* | [19] |
| NCU09830 | >gi\|1420383\|emb\|CAA99359.1\| PDR5  >gi\|927337\|gb\|AAB64846.1\| Pdr15p  >gi\|1431430\|emb\|CAA98831.1\| SNQ2 | ABC multidrug transporter | FGSC #11262 | *S. cerevisiae* | [19] |
| NCU04452 | >gi\|2326834\|emb\|CAA97877.1\| OYE3 | 12-oxophytodienoate reductase 1 | FGSC #11761 | *S. cerevisiae* | [19] |
| NCU06847 | >gi\|256271960\|gb\|EEU06979.1\| Tpo1p  >gi\|256269633\|gb\|EEU04915.1\| Tpo4p | major facilitator superfamily transporter | FGSC #12621 | *S. cerevisiae* | [19] |
| NCU06860 | >gi\|256271960\|gb\|EEU06979.1\| Tpo1p  >gi\|256269633\|gb\|EEU04915.1\| Tpo4p | MFS multidrug transporter | FGSC #12626 | *S. cerevisiae* | [19] |
| NCU08056 | >gi\|1420383\|emb\|CAA99359.1\| PDR5 >gi\|927337\|gb\|AAB64846.1\| Pdr15p  >gi\|1431430\|emb\|CAA98831.1\| SNQ2 | ABC drug exporter AtrF | FGSC #13048 | *S. cerevisiae* | [19] |
| NCU06077 | >gi\|256271960\|gb\|EEU06979.1\| Tpo1p  >gi\|256269633\|gb\|EEU04915.1\| Tpo4p | membrane transporter | FGSC #13560 | *S. cerevisiae* | [19] |
| NCU03358 | >gi\|1723933\|sp\|P53111.1\|ARI1_YEAST | ketoreductase | FGSC #14915 | *S. cerevisiae* | [19] |
| NCU00754 | >gi\|256271960\|gb\|EEU06979.1\| Tpo1p  >gi\|256269633\|gb\|EEU04915.1\| Tpo4p | multidrug resistant protein | FGSC #15939 | *S. cerevisiae* | [19] |
| NCU00306 | >gi\|256271960\|gb\|EEU06979.1\| Tpo1p | MFS multidrug transporter | FGSC #16036 | *S. cerevisiae* | [19] |
| NCU10009 | >gi\|1420383\|emb\|CAA99359.1\| PDR5  >gi\|927337\|gb\|AAB64846.1\| Pdr15p | ATP-binding cassette transporte | FGSC #16230 | *S. cerevisiae* | [19] |
| NCU03402 | >gi\|256269633\|gb\|EEU04915.1\| Tpo4p | membrane transporter | FGSC #16424 | *S. cerevisiae* | [19] |
| NCU07740 | >gi\|256269633\|gb\|EEU04915.1\| Tpo4p | hypothetical protein | FGSC #17901 | *S. cerevisiae* | [19] |
| NCU04161 | >gi\|1323513\|emb\|CAA97312.1\| YOR1 | multidrug resistance-associated protein 5 | FGSC #18978 | *S. cerevisiae* | [19] |
| NCU09580 | >gi\|256271960\|gb\|EEU06979.1\| Tpo1p  >gi\|256269633\|gb\|EEU04915.1\| Tpo4p | MSF membrane transporter | FGSC #20469 | *S. cerevisiae* | [19] |
| NCU04823 | >gi\|256272177\|gb\|EEU07174.1\| Adh6p | NADP-dependent alcohol dehydrogenase C | FGSC #16569 | *S. cerevisiae* | [56] |

**Table S5:** Inhibition in relative growth of different mutant strains in the presence of 60 mM of furfural, on solid media. The presented results were normalized to wild-type. The values and standard errors represent the average of at least three biological replicates.

| **Strain** | **Growth (%)** |
| --- | --- |
| T112 | 143.4±22.3 |
| Δ*ahd-2* | 47.3±8.9 |
| Δ*flr-1* | 62.4±5.11 |
| Δ*ncw-1* | 130.5±0.9 |
| Δ*cre-1* | 132.6±4.5 |
| P*tef*::*ahd-2* | 158.6±25.23 |
| ΔNCU02488 | 133.6±8.19 |
| T361 | 164.9±27.13 |
| P*cpc*::*flr-1* | 163.1±3.7 |
| ΔNCU01407 | 140.5±7.18 |

**Table S6:** Summary of genes identified in this study that are involved in tolerance to furfural.

| **Gene name** | **NCU** | **Function** | **log2(fold change)** | **Identified by** |
| --- | --- | --- | --- | --- |
| *cre-1* | NCU08807 | carbon catabolite regulation | NS | enrichmenet of carbohydrate metabolic process in RNA-seq |
| *ahd-2* | NCU00378 | aldehyde dehydrogenases | 1.02 | RNA-seq |
| *flr-1* | NCU05580 | membrane transporter  major facilitator superfamily | NS | hypothesis based |
|  | NCU02488 | hypothetical protein | NS | random mutagenesis |
| *ncw-1* | NCU05137 | non-anchored cell wall protein 1 | -5.40 | random mutagenesis |
|  | NCU01407 | zinc finger C3H1-type profile | NS | random mutagenesis |

**Table S7:** Strains of *N. crassa* used in this study.

| **Strain** | **Genotype** | **Source** |
| --- | --- | --- |
| Wild-type | *74-OR23-1 A* | FGSC #987 |
| Wild-type | *ORS-SL6 a* | FGSC #4200 |
| Δ*cre-1* | NCU08807 | [46] |
| ∆*ahd-2* | NCU00378 | FGSC #12919 |
| Ptef1-*ahd-2* | Ptef1-*ahd-2* -gfp | This study |
| *Δflr-1* | NCU05580 | FGSC #13741 |
| OE*flr-1* | *pCPC-1Δ2uorf::NCU05580* | This study |
| T112 | *hyg^R^* | This study |
| T361 | *hyg^R^* | This study |
| Δ*ncw-1* | NCU05137 A | FGSC #11682 |
| *Δ*NCU01407 | NCU01407 heterokaryon | FGSC #20117 |
| *Δ*NCU02488 | NCU02488 a | FGSC #17190 |

**Table S8:** Oligonucleotides used in this study.

| **Gene** | **Primer designation** | **Sequence (5′ --->3′)** | **Amplicon (bp)** | **Additional info** | **Reference** |
| --- | --- | --- | --- | --- | --- |
| β-tubulin | BT_92F | ATGCCTCCGGTGTGTACAATG | 79 | Gene expression | [71] |
|  | BT_171Rev | GTTGCCGGAAGCCTCGTT |  |  |  |
| NCU05580 | NCU05580_F1318 | TTGCCGGCCTGTTTATCTAC | 95 |  | This study |
|  | NCU05580_R1436 | GAAAGCCTCGAGCAGATACG |  |  |  |
| NCU02488 | 02488qPCR_353F | GGACCACAACTCCTCCTGAA | 86 |  |  |
|  | 02488qPCR_439R | CCTGCAGTTGACCCTTCATAG |  |  |  |
| NCU01407 | 01407qPCR_87F | TCGTCATACTGCCGTCGTTA | 77 |  |  |
|  | 01407qPCR_164R | TTTAGCGTAAAGGGGACGTG |  |  |  |
| pCPC-1 | pMP6-181F | TGTGAGCGGATAACAATTTCAC | 11561 | construction of OE*flr-1* |  |
|  | pMP6-11742R | ATGGGTACCTGAGAACATCTTGTTG |  |  |  |
| NCU05580 | NCU05580-25F linker | CAACAAGATGTTCTCAGGTACCCATTCAGGTTCAGACATCATCACAACAA | 2315 |  |  |
|  | NCU05580-2340R | TCAGATAAGGTCTCCATCTAAAACG |  |  |  |
|  | NCU05580-637F | CGGATTCGGTCTCTATCATATTTGC | 583 |  |  |
|  | NCU05580-54R | GTGAAATTGTTATCCGCTCACATATAGTCTGGACACCAGGGGTTGTT |  |  |  |
| NCU00378 | NCU00378_XbaI_F | TCTAGAATGAACCTCTTCCTCCG | 1701 | construction of Ptef1-*ahd-2* -gfp |  |
|  | NCU00378_PacI_R | TTAATTAATGCCGCCAGGTTTAC |  |  |  |
|  | Hphp | TGCAATAGGTCAGGCTCT | 1600 |  |  |
|  | NCU00378_3r | GCACTCTCTGGTATGTGTCC |  |  |  |
| NCU00378-gfp | NCU00378_F | CAAATCTGCACAGCCACTTC | 901 |  |  |
|  | pMF272_Rev | CGTCCTTGAAGAAGATGGTGC |  |  |  |
